# High incidence of postoperative silent venous thromboembolism in ulcerative colitis: a retrospective observational study

**DOI:** 10.1186/s12893-021-01250-y

**Published:** 2021-05-19

**Authors:** Norimitsu Shimada, Hiroki Ohge, Hiroki Kitagawa, Kosuke Yoshimura, Norifumi Shigemoto, Shinnosuke Uegami, Yusuke Watadani, Kenichiro Uemura, Shinya Takahashi

**Affiliations:** 1 National Hospital Organization Kure Medical Center and Chugoku Cancer Center, 3-1 Aoyama, Hiroshima 737-0023 Kure, Japan; 2 Department of Surgery, Hiroshima University, 1-2-3 Kasumi, Minami-ku, Hiroshima 734-8551 Hiroshima, Japan

**Keywords:** Ulcerative colitis, Prophylaxis, D-dimer, Ileal pouch, Portal–mesenteric venous thrombosis

## Abstract

**Background:**

The incidence of postoperative venous thromboembolism (VTE) is high in patients with inflammatory bowel disease. We aimed to analyze the incidence and predictive factors of postoperative VTE in patients with ulcerative colitis.

**Methods:**

Patients with ulcerative colitis who underwent colon and rectum surgery during 2010–2018 were included. We retrospectively investigated the incidence of postoperative VTE.

**Results:**

A total of 140 colorectal surgery cases were included. Postoperative VTE was detected in 24 (17.1 %). Portal–mesenteric venous thrombosis was the most frequent VTE (18 cases; 75 %); of these, 15 patients underwent total proctocolectomy (TPC) with ileal pouch–anal anastomosis (IPAA). In univariate analysis, VTE occurred more frequently in patients with neoplasia than in those refractory to medications (27.2 % vs. 12.5 %; *p* < 0.031). TPC with IPAA was more often associated with VTE development (28 %) than total colectomy (10.5 %) or proctectomy (5.9 %). On logistic regression analysis, TPC with IPAA, total colectomy, long operation time (> 4 h), and high serum D-dimer level (> 5.3 µg/mL) on the day following surgery were identified as predictive risk factors.

**Conclusions:**

Postoperative VTE occurred frequently and asymptomatically, especially after TPC with IPAA. Serum D-dimer level on the day after surgery may be a useful predictor of VTE.

## Background

Venous thromboembolism (VTE) is a well-known complication after colorectal surgery that leads to morbidity and mortality in hospitalized as well as discharged patients. In particular, patients with ulcerative colitis (UC) show a high incidence of VTE postoperatively. Many previous studies conducted in Western countries found that the risk of postoperative VTE in patients with UC was 2–3-fold higher than in patients with colorectal cancer, with VTE rates of 4.1–5.8 % [1, 2].

Current guidelines from the American Society of Colon and Rectal Surgeons recommend pharmacological prophylaxis with low-dose unfractionated heparin or low-molecular-weight heparin in patients with moderate and high risk for VTE following abdominal surgery. Patients with inflammatory bowel disease (IBD) are defined as having a high risk for VTE [3].

Despite many reports on postoperative VTE and guidelines that recommend pharmacological prophylaxis, it is not sufficiently administered in clinical practice. Possible reasons include the risk of postoperative bleeding from the remnant rectum with acute flare, epidural hematoma, and mainly low recognition of postoperative VTE by surgeons.

We suspected that more cases of postoperative VTE occurred in patients with UC in our clinical setting than described in the findings of the previous reports. As the data in the previous studies were extracted from large nationwide databases, they were considered reliable because there is less likelihood of bias in such large datasets. However, VTE with poor symptoms such as slight fever elevation or abnormal blood test data only may not have been included in these data. We therefore conducted a retrospective study of postoperative VTE in patients with UC in an IBD-specialized facility in Japan and reviewed all computed tomography (CT) scans that were performed postoperatively. The aim of the study was to clarify the incidence of postoperative VTE including portal–mesenteric venous thrombosis (PMVT) and identify predictive factors for VTE.

## Materials and methods

### Data collection and variables

**Fig. 1:**
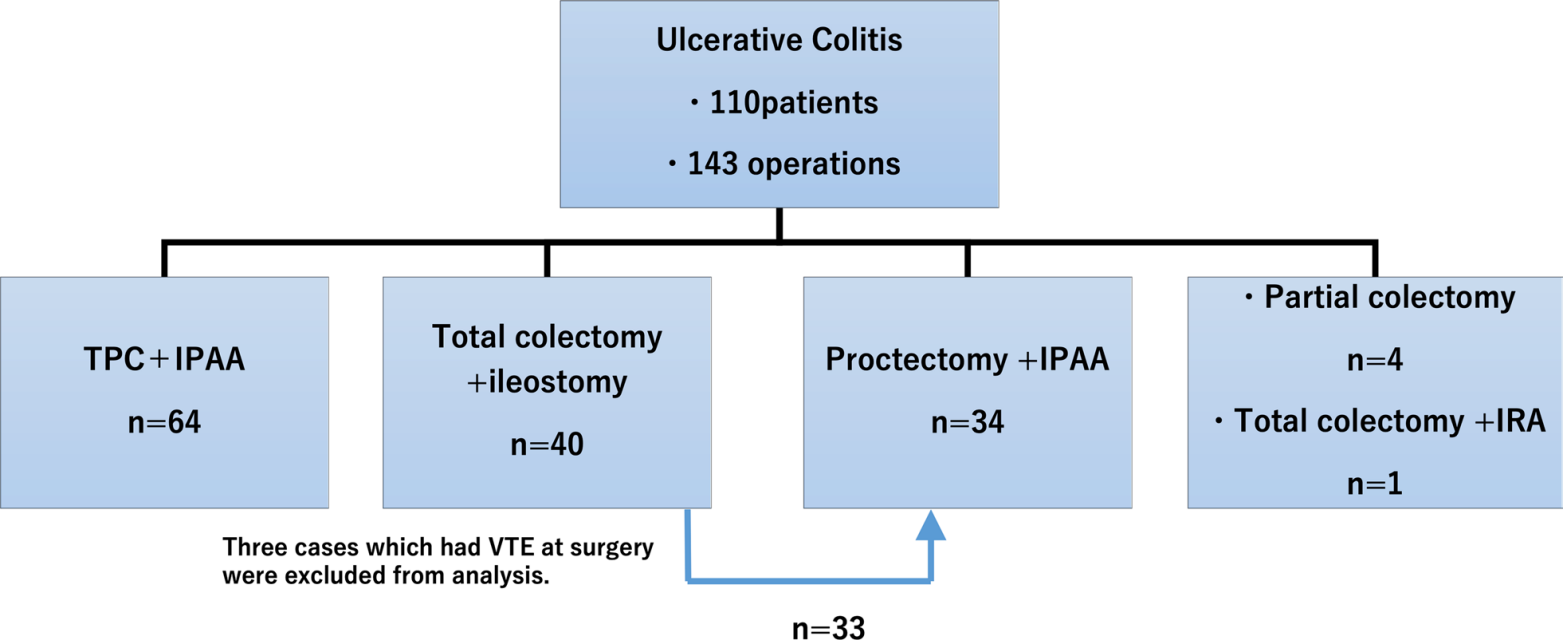
Schema of the enrolled patients and surgical procedures. We enrolled a total of 110 patients, including 143 surgical cases. After total colectomy, 33 patients underwent proctocolectomy with IPAA. Thirty-three of 34 patients who underwent proctectomy with IPAA had previously undergone total colectomy. Three cases with total colectomy were excluded from the analysis because they had preoperative venous thromboembolism. *IPAA* ileal pouch–anal anastomosis

We performed this retrospective observational study using a prospectively maintained database of patients with UC who underwent abdominopelvic bowel resection at Hiroshima University Hospital between January 2010 and December 2018. In total, we included 143 surgical cases among 110 patients. All patients were Asian Japanese. Restorative proctocolectomy and proctectomy were conducted with rectal mucosectomy and hand-sewn ileal pouch–anal anastomosis (IPAA) with a J pouch. Thirty-three patients with total colectomy (TC) underwent proctectomy with IPAA as a second surgery. When VTE was detected after TC, proctectomy was conducted after the thrombus had disappeared. Three patients who underwent TC were excluded because they showed preoperative VTE (Fig. 1). We reviewed the patients’ electronic medical charts to obtain information regarding patient characteristics, surgical procedures, and postoperative data. Emergency surgery was defined as unplanned surgery on admission.

The preoperative state of venous thrombosis was assessed using contrast-enhanced CT, with an image range from chest to pelvis, just prior to emergency surgery and within 4 weeks prior to elective surgery. Presence of postoperative VTE was assessed by contrast-enhanced CT examination from chest to pelvis as well as supplementary ultrasound examination. Preoperative therapy included use of steroid just prior to surgery, immunosuppressive agents within 1 week before surgery, and biological agents within 6 weeks prior to surgery.

All patients underwent early mobilization on the day following surgery and received mechanical VTE prophylaxis with compression stockings and an intermittent pneumatic compression device from the time of surgery until the start of walking. As pharmacological prophylaxis, low-molecular-weight heparin or unfractionated heparin was administered for 7 days after surgery. In our institution, we introduced pharmacological prophylaxis in 2015, except for cases with a risk of bleeding. Preoperative pharmacological prophylaxis was not administered to any patients.

## Outcome

The primary outcome was incidence of in-hospital VTE after abdominopelvic surgery. The secondary outcome was predictive risk factors for VTE.

In this study, VTE included pulmonary embolism (PE), deep vein thrombosis (DVT), and PMVT. DVT included thrombosis at the vena cava, internal jugular vein, subclavian vein, and femoral vein. The lower and upper limbs were not evaluated in the study.

### Statistical analysis

Analyses were conducted using JMP version 11 (SAS Institute Japan Ltd., Tokyo, Japan). Statistics are reported as median with interquartile range (IQR) for continuous variables and frequency with percentage for categorical variables. Normally distributed continuous variables were analyzed using the *t*-test, and nonnormally distributed continuous variables were analyzed with the nonparametric Wilcoxon’s rank-sum test. Fisher’s exact test was performed for categorical variables. Multivariable logistic regression analysis was conducted to identify predictive risk factors for surgical recurrence at the anastomosis site. Each analysis was performed using a two-sided 5 % significance level and 95 % confidence interval.

### Ethical considerations

This study was approved by the Hiroshima University Institutional Review Board (E-1636). Participants received information about the conduct of the research, including the purpose of the study, and were given the opportunity to decline participation in the study. Because the opt-out method can be used without informed consent from the patients, the information was published on the internet.

## Results

We included a total of 110 patients with UC in this study. The number of surgical cases was 143. The surgeries comprised 64 cases of TPC with ileal pouch-anal anastomosis (IPAA) as a two-stage procedure, 40 cases of TC with ileostomy, 34 cases of proctectomy with IPAA, 4 cases of partial colectomy, and one case of TC with ileorectal anastomosis. Thirty-three of the 34 patient who underwent proctectomy with IPAA had previously undergone TC with ileostomy as a three-stage procedure (Fig. 1).

Of the remaining 140 cases, postoperative VTE was detected in 24 cases (17.1 %) at 7 (5.2–9.7) days after surgery. Specifically, PMVT occurred in 18 cases (12.6 %), PE in 4 cases (2.8 %), femoral venous thrombus in 2 cases (1.4 %), subclavian vein thrombus in 1 case (0.7 %), and jugular vein thrombus in 1 case (0.7 %). Among these cases, two patients had multiple VTEs; one had PE + DVT and the other had PE + PMVT. PE was detected after TPC with IPAA in three cases and after TC in one case. Two patients with subclavian vein thrombus and jugular vein thrombus had central venous catheters after TC.

**Fig. 2:**
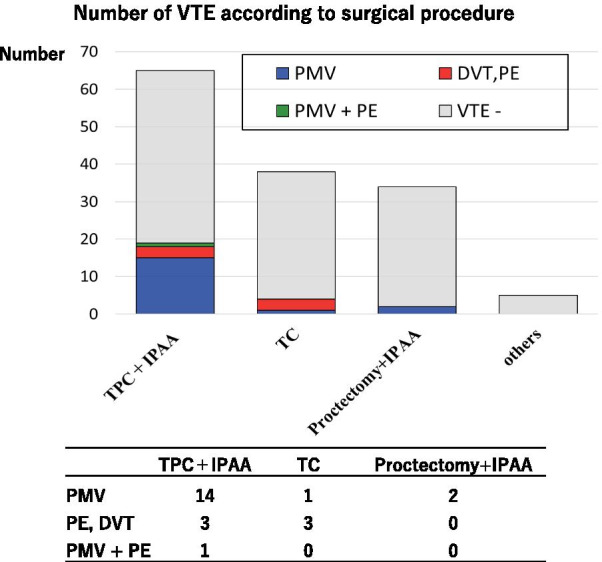
Numbers of VTE cases according to surgical procedures. The correlations between surgical procedures and thrombosis sites were determined. PMVT frequently occurred after TPC with IPAA. PE and DVT occurred more frequently in patients who underwent TC with acute flare-ups of UC. DVT included central vein and femoral vein thrombus. *TPC* total proctocolectomy, *IPAA* ileal pouch–anal anastomosis, *TC* total colectomy, *PMV* portal–mesenteric venous thrombosis

PMVT occurred most frequently, and 15 of these cases occurred after TPC with IPAA (Fig. 2). All 17 cases of liver portal vein thrombosis were detected in the right hepatic lobe, and 5 of these cases showed mesenteric venous thrombosis simultaneously. With respect to mesenteric venous thrombosis, five cases were in the inferior mesenteric vein, two cases were in the superior mesenteric vein, and one case was in the splenic vein. Patient backgrounds with VTE were not significantly different with those without VTE, except for the reason for surgery (Table 1).

**Table 1:** Patient characteristics according to venous thromboembolism

	Overall	VTE +	VTE −	*p* value
	N = 140	N = 24	N = 116	
*Gender, n (%)*				
Male	97 (69.3)	15 (62.5)	82 (70.6)	0.428
Female	43 (31.7)	9 (37.5)	34 (29.3)	
Age, median (range)	54 (41.2–63)	48 (38.2–57.5)	55.5 (42–64.7)	0.158
BMI, median (range)	21 (18.7–24.1)	21.8 (18.4–24)	20.5 (18.7–24.1)	0.81
ASA-PS ≧ 3, n (%)	8 (5.7)	1 (4.2)	7 (6.0)	0.719
Current smoking, n (%)	13 (9.3)	2 (8.3)	11 (9.48)	0.86
*Preoperative therapy, n (%)*				
Steroid	59 (42.1)	13 (54.2)	46 (39.6)	0.19
Immunosuppressive agents	48 (34.2)	9 (37.5)	39 (33.6)	0.72
Biological agents	20 (14.2)	3 (12.5)	17 (14.7)	0.78
*Comorbidity, n (%)*				
HT	20 (14.2)	5 (20.8)	15 (8.6)	0.31
HL	11 (7.9)	1 (4.2)	10 (8.6)	0.46
DM	15 (10.7)	0	15 (12.9)	0.073
*Indication, n (%)*				
Cancer/ dysplasia	44 (31.4)	12 (50)	32 (27.5)	
Refractory to medication	96 (68.5)	12 (50)	84 (87.5)	0.0313*

*VTE* venous thromboembolism, *BMI* body mass index, *ASA-PS* American Society of Anesthesiology-physical status, *HT* hypertension, *HL* hyperlipidemia, *DM* diabetes mellitus

*P < 0.05

Contrast-enhanced CT after surgery was conducted in 91 cases (65 %). The most frequent reasons for CT examinations were suspicion of pelvic abscess with fever elevation and white blood cell elevations, or outlet obstruction at the stoma site with abdominal pain and ileus. In the 24 cases with VTE, the reasons for CT examinations were AST/ALT elevation in 13 cases, white blood cell elevation in 10 cases, fever elevation in 6 cases, D-dimer elevation in 5 cases, and dyspnea in one case. Complications other than VTE were ileus in 6 cases (25 %) and pelvic abscess in 3 cases (4.2 %) (Table 2).

**Table 2:** Peri-operative results according to venous thromboembolism

	Overall	VTE +	VTE −	*p* value
	N = 140	N = 24	N = 116	
Procedure				
TPC IPAA, n (%)	64 (45.7)	18(75)	46 (39.7)	
TC, n (%)	38 (27.1)	4 (16.7)	34 (29.3)	
Proctectomy + IPAA, n (%)	34 (24.2)	2 (8.3)	32 (27.6)	
Other, n (%)	4 (2.8)	0	4 (3.4)	0.0144*
IPAA	96 (68.6)	20 (83.3)	76 (65.5)	0.087
Operative time, min.(range)	291 (203–383)	340 (255–476)	277 (181–355)	0.006*
Blood loss, ml (range)	300 (155–522)	326 (230–528)	275 (12–520)	0.1775
Complications				
Ileus, n (%)	28 (20)	6 (25)	22 (19)	0.57
Anastomosis leakage, n (%)	9 (6.4)	1 (4.2)	8 (6.9)	0.629
Pelvic abscess, n (%)	24 (17)	3 (12.5)	21 (18)	0.497
Emergency operation	36 (27.6)	5 (20.8)	31 (26.7)	0.5478
Prophylaxis with anticoagulant, n (%)	44 (31.4)	10 (41.7)	34 (29.3 %)	0.2956
D-dimer, µg/ml (range)				
POD1 D-dimer,	3.1 (2.2–5.8)	5.6 (2.7–8.9)	3 (2.2–4.7)	0.0218*
POD3 D-dimer	4.4 (3.1–8.25)	7 (3.5–10.6)	4.1 (3.1–7.7)	0.083
Peak D-dimer	8.85 (5.9–13)	10.1 (7.4–15.3)	8.7 (5.7–12)	0.072

*VTE* venous thromboembolism, *TPC* total proctocolectomy, *IPAA* ileal pouch–anal anastomosis, *TC* total colectomy, *POD* post-operative day

*P < 0.05

The rate of postoperative VTE prophylaxis with anticoagulant was 31.4 % in all patients, 41.7 % in the VTE (+) group, and 29.3 % in the VTE (−) group, respectively. Limited to the TPC + IPAA group, that was no significant difference in postoperative VTE prophylaxis between patients with VTE (9 cases; 50 %) and patients without VTE (19 cases; 41.3 %). With regard to surgical procedures, medical prophylaxis was performed in 28 cases (43.7 %) in the TPC + IPAA group, 6 cases (16.2 %) in the TC group, and 10 cases (29.4 %) in the proctectomy + IPAA group.

On univariate analysis, VTE occurred more frequently in patients with neoplasia (cancer or high-grade dysplasia) than in those refractory to medications (27.2 % vs. 12.5 %; *p* < 0.031). There were no significant differences in age, body mass index, American Society of Anesthesiology-physical status (ASA-PS) score ≥ 3, current smoking, preoperative therapy, and comorbidity between the VTE (+) group and VTE (−) group (Table 1). The perioperative results showed significant differences in the rates of VTE according to surgical procedures. Patients who underwent TPC with IPAA were more likely to develop VTE (28 %) than those who underwent TC (10.5 %) or proctectomy (5.9 %). Operation time in the VTE (+) group was significantly longer than that in the VTE (−) group (340 vs. 291 min, *p* = 0.006). Laboratory data indicated that serum D-dimer levels on the day following surgery were higher in the VTE (+) group (5.6 µg/mL vs. 3.1 µg/mL, *p* = 0.00218). D-dimer levels at day 3 after surgery were also higher in the VTE (+) group, but the difference was not significant (Table 2).

On logistic regression analysis, we identified TPC with IPAA, TC, long operation time (> 4 h), and serum D-dimer level > 5.3 mg/dL on the day following surgery as predictive risk factors for postoperative VTE in patients with UC (Table 3).

**Table 3:** Predictive risk factors in logistic regression analysis

		OR	95 % CI	*p* value
Procedure	Proctectomy + IPAA	Reference	−	−
	TC	45.88	1.44–2863	0.0304*
	TPC + IPAA	17.12	1.675–433.7	0.015*
Indication	Cancer/dysplasia	0.45	0.06–2.89	0.405
Operative time	(> 4 h)	28.84	1.23–1260	0.0358*
D-dimer at Day1	(> 5.3 µg/dl )	3.69	1.02–14.78	0.0452*

*OR* odds ratio, *CI* confidence interval, *TPC* total proctocolectomy, *IPAA* ileal pouch–anal anastomosis, *TC* total colectomy

*P < 0.05

## Discussion

In this study, postoperative VTE in patients with UC was detected in 24 cases (17.1 %) at a median of 7 days after surgery. PMVT was the most common type of VTE (18 cases; 12.8 %), and occurred most frequently after TPC with IPAA (23.4 %). Three predictive risk factors were detected in the logistic regression analysis (Table 3), namely elevated D-dimer on the day after surgery, surgical procedure (TPC with IPAA, TC), and long operation time (> 4 h).

IBD is a well-known independent risk factor for VTE, and patients with UC have a higher risk of VTE than those with Crohn’s disease [2, 4]. In patients with IBD, the risk of developing DVT and PE was 1.98- and 1.80-fold higher compared with other individuals, respectively, and the rates of postoperative VTE were 2.5–3.8 %, which was higher than the 2.4 % observed among patients with colorectal cancer [5–9]. The incidence rate of VTE in the present study (17.1 %) was higher than those in the previous reports. More than two-thirds of the cases had PMVT. When limited to DVT and PE, there were 7 cases (4.9 %), which was close to the frequencies reported in the previous studies.

CT examination after surgery was conducted for 91 cases (65 %), mostly because of abnormal laboratory data or fever elevation to rule out abscess, ileus, or VTE. In the 24 cases with VTE, only 5 cases were suspected of having VTE before CT examination, and the other 19 cases were unexpected. Frequent postoperative CT examinations for patients with mild symptoms made it possible to detect many VTE cases. Easy performance of CT may be related to the Universal Insurance System covering all people in Japan.

PMVT was most frequently detected after TPC with IPAA in the present study (23.4 %). There are few previous studies on PMVT. The reported rate of PMVT after colorectal surgery was 2.9–4.9 % [10, 11, 12]. IBD is a frequent cause of PMVT after abdominal surgery, with a reported rate of 8.3 % [13]. As a surgical procedure, restorative TPC with IPAA for patients with UC was identified as an independent predictor of PMVT, and the incidence rate was high at 5.8–10 % [14, 11, 15, 16]. In the procedure for ileal pouch construction and anal anastomosis, there were several factors that can promote thrombus formation in addition to the nature of IBD. These factors comprised long operation time, pelvic surgery in the lithotomy position, manipulation of mesenteric vessels, and traction of the superior mesentery vessels related to IPAA reconstruction. A dehydrated state caused by the covering ileostomy was also related to thrombosis, and occurred for all patients treated at our institution.

D-dimer elevation (> 5.3 mg/dL) on the day after surgery was identified as a predictive risk factor for postoperative VTE in this study, with sensitivity of 55 % and specificity of 78.5 %. D-dimer is not a specific marker for thrombus formation. Disease flares, bleeding, surgical invasion, and septic condition are also related to D-dimer elevation, even when the patients do not have VTE. Actually, 5 patients in the VTE (−) group had very high D-dimer levels (> 10 µg/mL), and all of them underwent emergency TC for bleeding or perforation with severe bowel inflammation. Therefore, D-dimer elevation is not a strong predictor of VTE, but can be easily used for suspicion of thrombus formation. It may be more useful for patients undergoing TPC or proctectomy whose preoperative condition is relatively stable compared with patients undergoing TC.

This study had several limitations. The study was designed retrospectively at a single institution, and the sample number was small. Variables that could be collected from the database were limited. Pharmacological prophylaxis was not administered to all patients, and the administration period was not defined. CT examination was conducted in all patients preoperatively, but not in all patients postoperatively, and the lower limbs were not examined for venous thrombus formation. Data for lower limb examinations using ultrasound imaging were not collected. Finally, the percentage of postoperative VTE prophylaxis with anticoagulant was only 31.4 %. This percentage is too small, given that the risks of VTE in patients with UC are well known. The current guideline states that strict administration of postoperative pharmacological prophylaxis is necessary, except for cases with a risk of bleeding from the remnant rectum [3].

## Conclusions

We found a high frequency of postoperative VTE, especially PMVT, in our retrospective dataset, particularly in one-quarter of patient who underwent TPC with IPAA. In most cases, the symptoms were mild, but may have the potential to become symptomatic with growth. It is important to recognize these findings, and to conduct strict anticoagulant therapy.

## Data Availability

The database used and analyzed during the current study are available from the corresponding author on reasonable request.

## Abbreviations

VTE: Venous thromboembolism
TPC: Total proctocolectomy
IPAA: Ileal pouch–anal anastomosis
UC: Ulcerative colitis
IBD: Inflammatory bowel disease
CT: Computed tomography
TC: Total colectomy
PE: Pulmonary embolism
DVT: Deep vein thrombosis
PMVT: Portal–mesenteric venous thrombosis
IQR: Interquartile range
